# Barriers and Facilitators That Influence HIV Pre-exposure Prophylaxis (PrEP)-Prescribing Behaviors Among Primary Care Providers in the Southern United States

**DOI:** 10.7759/cureus.66868

**Published:** 2024-08-14

**Authors:** Daryl O Traylor, Maithe Enriquez, Melva Thompson-Robinson, Mansoo Yu, Tina Bloom, Linda Bullock

**Affiliations:** 1 Public Health, A.T. Still University College of Graduate Health Sciences, Mesa, USA; 2 Basic Sciences, University of the Incarnate Word School of Osteopathic Medicine, San Antonio, USA; 3 Infectious Diseases, Sinclair School of Nursing, University of Missouri, Columbia, USA; 4 Public Health, School of Nursing, University of Nevada, Las Vegas, Las Vegas, USA; 5 Social Work and Public Health, University of Missouri, Columbia, USA; 6 School of Nursing, Notre Dame of Maryland University, Baltimore, USA; 7 Research, Sinclair School of Nursing, University of Missouri, Columbia, USA

**Keywords:** southern united states, pre-exposure prophylaxis, transtheoretical model, primary care providers, hiv prevention

## Abstract

The Southern United States (US) bears the highest burden of HIV prevalence in the country, disproportionately affecting African American communities. Despite the proven efficacy of pre-exposure prophylaxis (PrEP) in reducing HIV transmission, its uptake remains suboptimal in this region. This study aimed to identify factors influencing PrEP-prescribing behaviors among primary care providers (PCPs) in the Southern US through the application of the transtheoretical model of behavior change. A cross-sectional survey was conducted among PCPs in 10 Southern states to assess their PrEP-prescribing practices, barriers, and facilitators. The results indicate that non-White PCPs and those practicing in urban and suburban settings are more likely to prescribe PrEP. Key barriers include lack of training, perceived stigma, and systemic issues such as health insurance coverage and time constraints. Significant facilitators are access to prescribing resources, streamlined insurance procedures, and patient motivation. Targeted educational programs and policy changes to address these barriers can enhance PrEP uptake, thereby reducing HIV transmission in high-risk populations. The findings underscore the need for tailored interventions to support PCPs in integrating PrEP into routine care, ultimately contributing to better public health outcomes in the Southern US.

## Introduction

The Southern United States (US) bears the highest burden of HIV prevalence in the country, with African American communities experiencing a disproportionate impact [1]. According to epidemiological data, the Southern US accounts for over half of all new HIV diagnoses in the US, despite comprising only 38% of the national population [2,3]. Furthermore, the Southern US not only faces the highest HIV diagnosis rate but also the highest HIV-related mortality in the nation [3]. This region faces unique challenges, such as cultural barriers to receiving HIV care, lack of healthcare access, lower funding, stigma, and structural racism [3,4]. These alarming statistics underscore the urgent need for effective HIV prevention strategies in this region.

Building on this understanding, it is important to recognize that HIV has an affect on not just African American men [3-7]. The epidemic is primarily concentrated among African Americans, women, and rural residents in the Deep South [8]. Notably, Texas, Louisiana, Alabama, Mississippi, Florida, Tennessee, Arkansas, South Carolina, and North Carolina have experienced the highest rates of HIV diagnosis, mortality, and lowest survival rates as compared to the national data [5,6,8]. Factors contributing to this disparity include poverty, transportation access issues, stigma, lack of healthcare access, and the social determinants of health [9].

To address these disparities, pre-exposure prophylaxis (PrEP) has emerged as a highly effective strategy for preventing HIV transmission [10]. Clinical trials have demonstrated that when taken consistently, PrEP can reduce the risk of HIV infection by up to 99% in individuals at high risk [10]. Despite its proven efficacy, the PrEP uptake remains suboptimal, particularly in the Southern US where it is most critically needed [11]. Barriers to PrEP adoption include lack of awareness, stigma, and limited access to healthcare providers who are knowledgeable about PrEP [11-13].

Transitioning from efficacy to actual use, it is evident that despite the effectiveness of PrEP, access among African Americans in the Southern US remains low. A limited uptake is particularly observed among African American men who have sex with men (MSM) [14]. Barriers to PrEP use in this population include stigma, healthcare mistrust, poverty, and misinformation [15]. Geographic access to PrEP clinics is limited, especially in areas with higher poverty and historically underserved populations [16,17]. Moreover, awareness of PrEP is notably low among low-income African American cis/het women and transgender individuals [18].

Despite these barriers, there are promising interventions aimed at improving PrEP initiation and persistence among African American MSM [19]. Additionally, studies have shown that collaborations between public health departments and federally qualified health centers can significantly enhance access [20]. These collaborative efforts highlight the potential for systemic changes to bridge the gap in the PrEP uptake and effectively address the HIV epidemic in the Southern US.

Lack of primary care provider engagement in HIV screening and PrEP prescribing

A significant barrier to effective HIV prevention in the Southern US is the lack of engagement by primary care providers (PCPs) in HIV screening and PrEP prescribing. Despite the critical role that PCPs play in the healthcare system, many do not routinely offer HIV testing or discuss PrEP with their patients, particularly those from high-risk populations such as African Americans [21]. This lack of engagement can be attributed to several factors, including insufficient training, perceived stigma, and time constraints during patient consultations [22].

One major reason for the low engagement of PCPs in PrEP prescribing is a lack of adequate training and education on HIV prevention strategies. Many PCPs report feeling unprepared to discuss PrEP with their patients due to gaps in their knowledge and understanding of the medication's efficacy and usage guidelines [21]. This gap in education is further exacerbated by the limited inclusion of HIV prevention in medical school curricula and continuing medical education (CME) programs, leaving many PCPs without the necessary tools to effectively advocate for PrEP [23,24].

Additionally, perceived stigma and personal biases among healthcare providers can significantly hinder the adoption of PrEP-prescribing practices [22]. Some PCPs may hold stigmatizing views towards individuals at high risk of HIV, such as African American MSM and transgender individuals, which can lead to reluctance in offering PrEP as a preventative measure [25]. This stigma not only affects the willingness of PCPs to prescribe PrEP but also impacts the trust and communication between patients and providers, further discouraging patients from seeking PrEP [22].

Time constraints during patient consultations also pose a significant barrier to PrEP prescribing. Many PCPs operate under heavy workloads with limited time to spend on preventive health discussions. As a result, discussions about PrEP, which require a detailed conversation about HIV risk behaviors, medication adherence, and potential side effects, are often deprioritized or overlooked [22]. This issue is particularly pronounced in under-resourced areas of the Southern US, where healthcare providers may be stretched thin by high patient volumes and limited healthcare infrastructure [21].

Researching the phenomena of low PCP engagement in HIV screening and PrEP prescribing is critical for several reasons [26]. Understanding the barriers faced by PCPs can inform targeted interventions and educational programs designed to equip these providers with the knowledge and skills needed to effectively prescribe PrEP. Additionally, addressing these barriers can lead to improved patient-provider relationships, fostering a more supportive environment for discussing HIV prevention. Increasing the engagement of PCPs in PrEP prescribing may significantly enhance the overall uptake of PrEP, thereby reducing HIV transmission rates and improving public health outcomes in the Southern US [26].

Transtheoretical stages of change model and its application to PrEP-prescribing behavior

The transtheoretical model (TTM), developed by Prochaska and DiClemente in the late 1970s, is a widely utilized theoretical framework in health behavior change. The model posits that individuals move through a series of stages when modifying behavior: precontemplation, contemplation, preparation, action, and maintenance [25]. Each stage represents a different level of readiness to change, and understanding these stages can provide insights into how to effectively encourage behavior modification.

Stages of Change

In the precontemplation stage, individuals are not considering change and may be unaware of the need for change. Moving to the contemplation stage, individuals recognize the need for change and begin to consider it, but have not yet committed to taking action. In the preparation stage, individuals are planning to act soon and may start making small changes. During the action stage, individuals have recently begun to implement the behavior change. Finally, in the maintenance stage, individuals sustain the behavior change over time and work to prevent relapse [25].

Application to PrEP-Prescribing Behavior

The TTM may be particularly useful as a predictor for the PrEP-prescribing behavior among PCPs due to its focus on the readiness to change [25]. In the context of HIV prevention, the model can help identify where PCPs are in terms of their willingness and ability to prescribe PrEP.

In the precontemplation stage, PCPs may not consider PrEP prescribing relevant or necessary and may lack awareness about the efficacy of PrEP or have misconceptions about its use. In the contemplation stage, PCPs start to acknowledge the importance of PrEP but have not yet committed to integrating it into their practice, and they may seek more information or begin to consider how it could benefit their patients. During the preparation stage, PCPs plan to start prescribing PrEP and may attend training sessions or seek out resources to understand the prescribing process better. In the action stage, PCPs actively prescribe PrEP and discuss it with eligible patients, integrating PrEP discussions into routine care and building confidence in managing PrEP patients. Finally, in the maintenance stage, PCPs have incorporated PrEP prescribing into their regular practice and continue to update their knowledge and skills while addressing any barriers that could prevent sustained PrEP use among patients.

Decisional balance 

Understanding Decisional Balance

Decisional balance, a core construct of the TTM of behavior change, refers to the process of weighing the pros and cons of changing a behavior [26]. This concept is essential for understanding how individuals make decisions about adopting new behaviors, particularly in the context of health interventions. Decisional balance involves evaluating the perceived benefits (pros) and costs (cons) associated with a particular action, which influences an individual's readiness to change [27].

In the TTM, decisional balance shifts as individuals progress through the stages of change.

Application to PCP PrEP Prescribing

In the context of PCP prescribing of PrEP for HIV prevention, decisional balance can be a crucial factor influencing their prescribing behavior. The following aspects illustrate how decisional balance may be applied.

Pros of prescribing PrEP include its efficacy in preventing HIV. PCPs recognize the high efficacy of PrEP in reducing HIV transmission, especially in high-risk populations. Additionally, prescribing PrEP contributes to broader public health goals by reducing the overall incidence of HIV. Providers also understand that offering PrEP can significantly improve the health and safety of their patients at risk of HIV.

On the other hand, some PCPs may perceive cons to prescribing PrEP. Many PCPs may feel unprepared to prescribe PrEP due to insufficient training or gaps in their knowledge about its use and guidelines. Concerns about stigma associated with HIV and PrEP may deter PCPs from initiating discussions about PrEP with their patients. Additionally, adherence issues can be a concern, as PrEP requires strict compliance to be effective, and inconsistent use may lead to drug resistance. The need for regular monitoring, such as renal function tests, can strain limited resources and time. There is also apprehension about unintended behavioral consequences, where patients may engage in riskier behaviors under the belief that PrEP offers complete protection, potentially increasing other sexually transmitted infection (STI) rates. Furthermore, cultural and ethical considerations, patient selection challenges, and potential language barriers can make discussions about PrEP difficult. PCPs may also have concerns about the long-term effects of PrEP, regulatory and legal implications, and limited patient awareness of or demand for PrEP, making it challenging to justify the investment of time and resources. These factors, coupled with issues such as a lack of health insurance coverage and time constraints during consultations, may discourage PCPs from prescribing PrEP.

Shifting the Balance

To enhance PrEP prescribing among PCPs, interventions can be designed to shift the decisional balance by increasing the perceived benefits and reducing the perceived costs. This can be achieved through targeted education and training, providing comprehensive training programs that equip PCPs with the necessary knowledge and skills to prescribe PrEP confidently. Addressing stigma is another critical aspect, which can be tackled by implementing initiatives to reduce stigma associated with HIV and PrEP through awareness campaigns and fostering a supportive clinical environment. Streamlining processes is also essential; insurance companies should develop simplified health insurance prior authorization procedures, and providing resources such as PrEP-prescribing guidelines may reduce the systemic barriers faced by PCPs. Finally, emphasizing the benefits, including highlighting the public health impact and patient benefits of PrEP, can reinforce the pros of prescribing it, encouraging PCPs to integrate PrEP into routine care.

By understanding and leveraging the concept of decisional balance, healthcare systems can design effective strategies to promote PrEP prescribing among PCPs, ultimately contributing to better HIV prevention outcomes in high-risk populations.

Benefits of using the TTM for PrEP prescribing

Utilizing the TTM to understand and predict PrEP-prescribing behavior offers several advantages. Tailored interventions can be designed to target PCPs at different stages of readiness, making them more effective. For instance, educational materials and training sessions can be customized to address the specific needs and concerns of PCPs at each stage. Additionally, the model recognizes that behavior change is a gradual process. By acknowledging small steps and incremental progress, it can help maintain motivation and reduce the likelihood of relapse. Furthermore, the TTM provides a comprehensive framework that considers both individual and systemic factors influencing behavior change. This holistic approach can lead to more sustainable improvements in PrEP-prescribing practices.

By identifying the stage at which a PCP is operating, targeted interventions can be designed to move them through the stages towards the action and maintenance phases. For instance, those in the precontemplation stage might benefit from educational campaigns that highlight the high HIV burden in the Southern US and the effectiveness of PrEP. PCPs in the contemplation stage might need more detailed information on how to discuss PrEP with patients and address common concerns. Those in the preparation stage could benefit from practical training sessions that build their confidence and competence in prescribing PrEP.

Need for the study

The need for this study is critical due to the persistently low rates of PrEP prescribing by PCPs in the Southern US, despite the region's high HIV prevalence and significant risk among African American communities. The current literature inadequately addresses the specific barriers and facilitators that influence PCPs' prescribing practices in this context. By identifying these factors, the study aims to inform targeted interventions and educational programs that can enhance the PrEP uptake and ultimately reduce HIV transmission in this high-risk population. Understanding and addressing these gaps is essential for improving health equity and achieving more effective HIV prevention strategies.

## Materials and methods

Research design

This cross-sectional study utilized a convenience sample to examine factors influencing the PrEP-prescribing behaviors of primary care providers in the Southern United States. The study aimed to provide a quantitative assessment of PCPs' personal and professional variables aligned with the transtheoretical stages of change model to predict PrEP-prescribing practices. The outcome variables of interest were the stage of adoption of PrEP prescribing and whether or not PrEP was prescribed.

Research questions

The research questions for the study were as follows: (1) What are the relationships between PCP personal and practice variables and the stage of adoption, using the TTM, of PrEP prescribing? (2) What PCP personal and practice characteristics predict prescribing or not prescribing PrEP? (3) What is the relationship between the TTM decisional balance construct and the TTM stages of change for PrEP prescribing? (4) Does PCPs’ TTM decisional balance predict prescribing or not prescribing PrEP?

Participant recruitment

Participants were recruited using social media and email due to the COVID-19 lockdowns, between March 7, 2021, and May 8, 2021, following approval from the University of Missouri, Columbia's Institutional Review Board (no. 2056192). Invitations were extended to PCPs practicing in the Southern US, encouraging those interested to participate in an online survey hosted on Qualtrics® (Provo, UT, USA). To maintain provider confidentiality, no identifying information or internet protocol addresses were collected.

Eligibility Criteria

To be eligible for the study, participants had to provide consent to participate, be 18 years of age or older, be a licensed physician, advanced practice registered nurse, or physician assistant, be currently practicing primary care medicine, and practicing in one of the following states: Arkansas, Alabama, Georgia, Florida, Louisiana, Mississippi, North Carolina, South Carolina, Tennessee, or Texas. Participants who did not meet these criteria were excluded from the study.

Data collection procedure

Upon accessing the survey link, participants were directed to an information page detailing the study's purpose and informed consent. After consenting, they were assessed for eligibility through a series of screening questions. Eligible participants then completed the survey, which included questions about their personal demographics, practice characteristics, and PrEP-prescribing behaviors.

Survey Instrument

The survey was adapted from the HIV PrEP survey used by Terndrup et al. [27], incorporating the TTM staging algorithm. Permission was sought and granted from Terndrup et al. to use the survey, though the validity and reliability of the instrument were not established. The survey included five screening and consent questions, one TTM staging question, two TTM decisional balance questions regarding barriers and facilitators to PrEP prescribing, and 21 questions on provider demographics and practice variables. The survey assessed factors such as the geographic location of practice, years of practice, and whether African American patients were seen and screened for PrEP (see the Appendices).

Sample size and power analysis

A power analysis using G*Power 3.1.9.4 (Heinrich Heine University Düsseldorf, Germany) was conducted to determine the appropriate sample size for logistic regression analysis. Based on an odds ratio of 2 for a medium effect size, an alpha of .05, and a power of .80, the desired sample size was 219. To account for potential incomplete surveys, 300 participants were targeted.

Data analysis

Data was analyzed using IBM SPSS Statistics, version 26 (IBM Corp., Armonk, NY). The dataset was screened for missing responses, patterns, and inconsistencies. Frequency distributions and summary statistics were calculated to detect outliers. Ordinal logistic regression was used to analyze the relationships between multiple independent variables (e.g., age, race/ethnicity, gender, sexual orientation) and the ordinal dependent variable (stage of PrEP prescribing). Missing data was handled using pairwise exclusion.

## Results

A total of 330 responses were submitted to the online survey. One hundred and seven responses were removed because the participants did not finish all sections of the survey, leaving a total of 223 participants included in the final dataset. All remaining missing responses to individual questions were handled using pairwise exclusion.

Table 1 outlines the demographic profile of the study participants. The participants had an average age of 44.04 years (SD = 10.81). A majority were men (n = 129, 57.8%), with most identifying as heterosexual (n = 180, 80.7%). The predominant racial identity was White (n = 113, 50.7%), and the majority indicated they were not Hispanic (n = 182, 81.6%). The largest group of participants practiced in Texas (n = 118, 52.9%) and in urban settings (n = 151, 67.7%). The most common profession among the participants was that of physicians (n = 149, 66.8%), with the highest educational attainment being having a medical doctor (n = 100, 44.8%). On average, participants had 13.65 years of experience in primary care (SD = 9.77).

**Table 1:** Sample demographics

Variable	Frequency	Percentage
Practicing state		
Arkansas	11	4.9
Alabama	7	3.1
Georgia	30	13.5
Florida	10	4.5
Louisiana	6	2.7
Mississippi	9	4.0
North Carolina	8	3.6
South Carolina	14	6.3
Tennessee	6	2.7
Texas	118	52.9
Multiple	3	1.3
No response	1	0.4
Geographic area		
Urban	151	67.7
Suburban	31	13.9
Rural	35	15.7
No response	6	2.7
Gender		
Male	129	57.8
Female	85	38.1
Genderqueer/non-conforming	4	1.8
Transgender	2	0.9
Prefer not to answer	2	0.9
No response	1	0.4
Sexual orientation		
Heterosexual	180	80.7
Gay	17	7.6
Lesbian	12	5.4
Bisexual	4	1.8
Other	4	1.8
Prefer not to answer	4	1.8
No response	2	0.9
Race		
American Indian or Alaskan Native	4	1.8
Asian	15	6.7
Black or African American	48	21.5
Native Hawaiian or Pacific Islander	2	0.9
White	113	50.7
Multiracial or multiple selections	18	8.1
Other	4	1.8
Prefer not to answer	19	8.5
Hispanic		
Yes	38	17.0
No	182	81.6
No response	3	1.3
Type of practitioner		
Physician	149	66.8
Advanced Practice Registered Nurse	51	22.9
Physician Assistant	22	9.9
No response	1	0.4
Highest degree completed		
Doctor of Osteopathic Medicine (DO)	49	22.0
Medical Doctor (MD)	100	44.8
Master of Science - Nursing (MSN) or equivalent	30	13.5
Doctor of Nursing Practice (DNP)	20	9
Master of Science - Physician Assistant (MSPA) or equivalent	19	8.5
Doctor of Medical Science - Physician Assistant (DMSPA) or equivalent	3	1.3
No response	2	0.9

PrEP-prescribing practices and patient demographics: insights through the TTM

Table 2 presents descriptive statistics concerning participants' PrEP practices and their patients. The data indicates that participants were primarily at either the lowest (n = 72, 32.3%) or highest (n = 69, 30.9%) stages of PrEP prescribing. A significant portion of participants did not regularly screen African American patients for HIV (n = 130, 58.3%), assess their need for PrEP (n = 149, 66.8%), prescribe PrEP (n = 182, 82.1%), or refer them for PrEP prescriptions (n = 185, 83.0%). Over half of the respondents reported that they had never written a PrEP prescription for an African American patient (n = 128, 57.4%), and 70.4% had not received PrEP training (n = 157). Despite this, most participants were familiar with PrEP prior to the survey (n = 181, 81.2%). Additionally, most participants indicated that 50% or fewer of their patients were African American (n = 178, 79.8%).

**Table 2:** PrEP practices and patient characteristics PrEP, pre-exposure prophylaxis

Variable	Frequency	Percentage
Stage of PrEP prescribing		
Does not prescribe and does not intend to within 6 months	72	32.3
Does not prescribe but intends to within 6 months	37	16.6
Does not prescribe but intends to within 30 days	18	8.1
Has prescribed for less than 6 months	27	12.1
Has prescribed for more than 6 months	69	30.9
Do you routinely screen your African American patients for HIV?		
Yes	92	41.3
No	130	58.3
No response	1	0.4
Do you routinely screen your African American patients for PrEP need?		
Yes	74	33.2
No	149	66.8
Have you ever written a prescription for PrEP for an African American patient?		
Yes	95	42.6
No	128	57.4
Do you routinely write PrEP prescriptions for your African American patients?		
Yes	40	17.9
No	183	82.1
Have you ever referred an African American patient for a PrEP prescription?		
Yes	92	41.3
No	130	58.3
No response	1	0.4
Do you routinely refer African American patients for PrEP prescriptions?		
Yes	36	16.1
No	185	83.0
No response	2	0.9
Have you received specific training on PrEP?		
Yes	65	29.1
No	157	70.4
No response	1	0.4
Before this survey, had you ever heard of PrEP?		
Yes	181	81.2
No	41	18.4
No response	1	0.4
% of patients who are African American		
0%	4	1.8
1%-25%	97	43.5
26%-50%	81	36.3
51%-75%	21	9.4
76%-100%	16	7.2
No response	4	1.8
% of patients who are White		
0%	3	1.3
1%-25%	51	22.9
26%-50%	76	34.1
51%-75%	53	23.8
76%-100%	34	15.2
No response	6	2.7
% of patients who are American Indian or Alaskan Native		
0%	68	30.5
1%-25%	101	45.3
26%-50%	2	0.9

Chi-square tests of independence were performed to assess whether the stage of change regarding PrEP prescribing and the act of prescribing PrEP varied based on the participants' self-reported proportion of African American patients. The participants were categorized into two groups: those with “50% or less” African American patients and those with “more than 50%” African American patients. Tables 3, 4 present the cross-tabulations of these variables. The chi-square test for the PrEP stage of change was significant, χ^2^(4) = 17.27, p = .002, suggesting that participants with a higher proportion of African American patients were more likely to be in an advanced stage of change. Similarly, the chi-square test for PrEP prescription was significant, χ^2^(1) = 16.41, p = .001, indicating that participants with a greater proportion of African American patients were more likely to have prescribed PrEP.

**Table 3:** Cross-tabulation of percentage of African American patients versus pre-exposure prophylaxis (PrEP) stage of change

Percentage of African American patients	Stage 1, n (%)	Stage 2, n (%)	Stage 3, n (%)	Stage 4, n (%)	Stage 5, n (%)
50% or less	66 (36%)	33 (18%)	15 (8%)	10 (11%)	48 (26%)
More than 50%	4 (11%)	3 (8%)	3 (8%)	7 (19%)	20 (54%)

**Table 4:** Cross-tabulation of percentage of African American patients versus pre-exposure prophylaxis (PrEP) prescription

Percentage of African American patients	Prescribed PrEP, n (%)	Never prescribed PrEP, n (%)
50% or less	67 (37%)	115 (63%)
More than 50%	27 (73%)	10 (27%)

Facilitators and barriers to PrEP prescribing

The study assessed the factors influencing decisions to prescribe PrEP among participants, using a Likert scale ranging from 1 (not important) to 5 (extremely important). Table 5 presents the mean ratings and standard deviations for both facilitators and barriers identified. The highest-rated factor, averaging a score of 4.70 (SD = 0.80), was “lack of insurance coverage”. Conversely, “clinic in-service PrEP training” received the lowest average rating of 1.83 (SD = 1.30).

**Table 5:** Descriptive statistics for facilitators and barriers to PrEP prescribing PrEP, pre-exposure prophylaxis

Variable	M	SD
PrEP training during residency	2.35	1.57
Staff or providers who are knowledgeable about PrEP provision	3.27	1.45
Access to resources such as PrEP prescription guidelines and protocols	3.63	1.53
On-site support	2.67	1.47
Clinic in-service PrEP training	1.83	1.30
Knowledge of PrEP's efficacy	2.85	1.70
Patient motivation or "buy in"	4.25	1.19
Peers who prescribe PrEP	3.18	1.66
Patient access to financial incentives that would lower the cost of PrEP	2.77	1.51
Streamlined insurance prior authorization procedures	4.32	1.25
Lack of provider training/education regarding PrEP	4.27	1.20
Lack of clinic leadership support regarding PrEP	3.10	1.42
Lack of clinical guidelines/protocols for prescribing/monitoring PrEP	3.87	1.38
Clinic and lab monitoring requirements	2.73	1.41
Staffing time constraints related to risk reduction and PrEP adherence counseling	4.29	1.07
Lack of insurance coverage and out-of-pocket patient costs for PrEP	4.70	0.80
Likelihood of low adherence to PrEP	3.32	1.51
Likelihood of developing HIV resistance	2.27	1.49
Patients may engage in riskier behavior while on PrEP	3.25	1.55
Insufficient evidence of PrEP's effectiveness	1.98	1.37

Determinants of PrEP-prescribing practices and stages of change among PCPs

*Research Question 1:* *What Is the Relationship Between Personal and Practice Variables and the Stage of Change of PrEP Prescribing?*

To address Research Question 1, an ordinal logistic regression analysis was performed. The analysis included age, race/ethnicity, gender, and sexual orientation as personal variables, and type of primary care practitioner (physician, nurse practitioner, physician assistant), years of practice, practice setting (urban, suburban, rural), and receipt of PrEP training as practice variables. The dependent variable was the stage of change for PrEP prescribing, categorized into five ordinal levels indicating increasing adoption stages. Multicollinearity was assessed using variance inflation factors (VIFs), all of which were below 10, suggesting no severe multicollinearity.

The overall model was statistically significant, χ^2^(17) = 170.77, p < .001, indicating that the combined predictors significantly predicted the stage of change. Table 6 presents the individual regression coefficients. Race emerged as a significant predictor; non-White participants were 3.97 times more likely to be in a higher stage of change (p < .001). Practice setting also significantly influenced the stage of change, with urban (OR = 8.65, p < .001) and suburban settings (OR = 6.14, p = .004) associated with higher stages of change. Additionally, having received PrEP training significantly predicted a higher stage of change (OR = 18.41, p < .001).

**Table 6:** Ordinal logistic regression predicting the pre-exposure prophylaxis (PrEP) stage of prescribing

Variable	Estimate	Standard error	Wald's statistic	Significance	Odds ratio
Age	-0.04	0.03	1.97	.160	0.96
Years of practicing	0.01	0.03	0.18	.670	1.01
Male	0.61	2.10	0.09	.771	1.84
Female	0.92	2.09	0.19	.659	2.51
Genderqueer	1.04	2.16	0.23	.629	2.83
Transgender	-2.30	2.29	1.01	.316	0.10
Non-White	1.38	0.35	15.40	< .001	3.97
Hispanic	-0.06	0.41	0.02	.890	0.94
Heterosexual	-0.08	1.54	0.00	.956	0.92
Gay	2.27	1.70	1.79	.181	9.69
Lesbian	0.45	1.60	0.08	.777	1.57
Bisexual	0.95	1.87	0.26	.611	2.59
Physician	0.06	0.51	0.01	.912	1.06
Advanced Practice Registered Nurse	0.31	0.61	0.25	.618	1.36
Urban	2.16	0.54	16.02	< .001	8.65
Suburban	1.82	0.63	8.22	.004	6.14
Had PrEP training	2.91	0.41	50.19	< .001	18.41

*Research Question 2:* *Do Primary Care Providers’ Personal and Practice Variables Predict Prescribing or Not Prescribing PrEP to African Americans Residing in the Southern US?*

To address the second research question, a binary logistic regression analysis was performed. This analysis utilized the same personal and practice variables that were employed for the first research question. The final independent variables included in the model were selected through the forward entry (conditional) method. The dependent variable was whether the participant prescribed PrEP (categorized as prescribed or not prescribed).

The overall model was statistically significant, χ^2^(8) = 136.34, p < .001, demonstrating that the predictors collectively had a significant impact on PrEP prescription. Therefore, the null hypothesis was rejected. Table 7 presents the individual regression coefficients for the final model. Race emerged as a significant predictor, with participants who did not identify as White being 5.86 times more likely to prescribe PrEP (p < .001). Sexual orientation was also a significant predictor, with participants identifying as gay (OR = 359.51, p = .002), lesbian (OR = 19.24, p = .034), and bisexual (OR = 62.75, p = .030) showing a higher likelihood of prescribing PrEP. Additionally, the practice setting was significant, indicating that participants in urban settings (OR = 11.36, p = .008) were more likely to prescribe PrEP. Furthermore, PrEP training was a significant predictor, with those having received training being 40.26 times more likely to prescribe PrEP (p < .001).

**Table 7:** Binary logistic regression predicting pre-exposure prophylaxis (PrEP) prescription

Variable	Estimate	Standard error	Wald's statistic	Significance	Odds ratio
Non-White	1.77	0.48	13.58	< .001	5.86
Heterosexual	2.11	1.17	3.22	.073	8.21
Gay	5.89	1.93	9.28	.002	359.51
Lesbian	2.96	1.40	4.47	.034	19.24
Bisexual	4.14	1.90	4.74	.030	62.75
Urban	2.43	0.92	6.98	.008	11.36
Suburban	1.81	1.04	3.01	.083	6.08
Had PrEP training	3.70	0.60	37.56	< .001	40.26

*Research Question 3:* *What Is the Relationship Between TTM Decisional Balance and the Stage of Change of PrEP Prescribing?*

To address Research Question 3, an ordinal logistic regression analysis was conducted. In this analysis, the independent variables consisted of individual survey items that assessed the importance of specific facilitators and barriers to PrEP prescribing (based on the TTM decisional balance). The dependent variable was the stage of change for PrEP prescribing. Multicollinearity was evaluated by calculating VIFs, and all VIFs were below 10, indicating an absence of severe multicollinearity.

The overall model was statistically significant, χ^2^(20) = 135.78, p < .001, demonstrating that the predictors collectively had a significant impact on the stage of change. Consequently, the null hypothesis was rejected. Table 8 presents the individual regression coefficients for the model. Significant positive predictors included access to resources (OR = 1.70, p < .001), streamlined insurance (OR = 1.52, p = .010), and lack of insurance coverage (OR = 1.99, p = .004), indicating that participants who rated these factors as more important were more likely to be in an advanced stage of change. Conversely, significant negative predictors were lack of provider training (OR = 0.67, p = .020), likelihood of low adherence (OR = 0.66, p = .018), and likelihood of HIV resistance developing (OR = 0.64, p = .012), indicating that participants who rated these factors as more important were less likely to be in a higher stage of change.

**Table 8:** Ordinal logistic regression predicting the pre-exposure prophylaxis (PrEP) stage of prescribing

Variable	Estimate	Standard error	Wald's statistic	Significance	Odds ratio
PrEP training during residency	-0.04	0.11	0.14	.705	0.96
Staff or providers who are knowledgeable about PrEP provision	0.25	0.14	3.24	.072	1.28
Access to resources such as PrEP prescription guidelines and protocols	0.53	0.14	14.02	< .001	1.70
On-site support	-0.02	0.12	0.03	.853	0.98
Clinic in-service PrEP training	-0.08	0.16	0.28	.597	0.92
Knowledge of PrEP's efficacy	0.07	0.15	0.21	.650	1.07
Patient motivation or "buy in"	0.22	0.16	1.85	.174	1.25
Peers who prescribe PrEP	0.00	0.12	0.00	.984	1.00
Patient access to financial incentives that would lower the cost of PrEP	0.14	0.14	1.02	.312	1.15
Streamlined insurance prior authorization procedures	0.42	0.16	6.72	.010	1.52
Lack of provider training/education regarding PrEP	-0.40	0.17	5.40	.020	0.67
Lack of clinic leadership support regarding PrEP	-0.26	0.14	3.50	.061	0.77
Lack of clinical guidelines/protocols for prescribing/monitoring PrEP	-0.16	0.14	1.17	.279	0.86
Clinic and lab monitoring requirements	0.06	0.15	0.15	.697	1.06
Staffing time constraints related to risk reduction and PrEP adherence counseling	-0.03	0.19	0.03	.853	0.97
Lack of insurance coverage and out-of-pocket patient costs for PrEP	0.69	0.24	8.12	.004	1.99
Likelihood of low adherence to PrEP	-0.41	0.17	5.63	.018	0.66
Likelihood of developing HIV resistance	-0.45	0.18	6.34	.012	0.64
Patients may engage in riskier behavior while on PrEP	-0.09	0.15	0.35	.554	0.91
Insufficient evidence of PrEP's effectiveness	-0.07	0.18	0.13	.715	0.94

Research Question 4: Does a Primary Care Provider’s TTM Decisional Balance Predict Prescribing or Not Prescribing PrEP to African Americans Residing in the Southern US?

To address Research Question 4, a binary logistic regression analysis was performed. The independent variables in this analysis were the specific survey items concerning the perceived importance of facilitators and barriers to PrEP prescribing, which were used in Research Question 3 (reflecting the TTM decisional balance). The final set of independent variables included in the model was selected using the forward entry (conditional) method. The dependent variable was whether the participant prescribed PrEP (yes or no).

The final model was statistically significant, χ^2^(9) = 131.51, p < .001, indicating that the predictors collectively had a significant impact on the likelihood of prescribing PrEP, leading to the rejection of the null hypothesis. Table 9 provides the individual regression coefficients for the final model. Significant positive predictors included knowledgeable staff (OR = 1.51, p = .033), access to resources (OR = 1.85, p = .001), patient motivation (OR = 2.19, p = .007), patient access to financial incentives (OR = 1.97, p < .001), and lack of insurance coverage (OR = 2.74, p = .002), suggesting that participants who rated these factors as more important were more likely to prescribe PrEP. Conversely, significant negative predictors were lack of provider training (OR = 0.43, p = .003), lack of clinical leadership (OR = 0.65, p = .018), likelihood of low adherence (OR = 0.53, p = .001), and likelihood of developing HIV resistance (OR = 0.43, p < .001), indicating that participants who rated these factors as more important were less likely to prescribe PrEP.

**Table 9:** Binary logistic regression predicting pre-exposure prophylaxis (PrEP) prescription

Variable	Estimate	Standard error	Wald's statistic	Significance	Odds ratio
Staff or providers who are knowledgeable about PrEP provision	0.41	0.19	4.55	.033	1.51
Access to resources such as PrEP prescription guidelines and protocols	0.61	0.18	11.93	.001	1.85
Patient motivation or "buy in"	0.78	0.29	7.20	.007	2.19
Patient access to financial incentives that would lower the cost of PrEP	0.68	0.18	14.12	< .001	1.97
Lack of provider training/education regarding PrEP	-0.86	0.29	8.92	.003	0.43
Lack of clinic leadership support regarding PrEP	-0.43	0.18	5.57	.018	0.65
Lack of insurance coverage and out-of-pocket patient costs for PrEP	1.01	0.33	9.29	.002	2.74
Likelihood of low adherence to PrEP	-0.64	0.20	10.36	.001	0.53
Likelihood of developing HIV resistance	-0.84	0.22	14.78	< .001	0.43

## Discussion

The findings from this study provide significant insights into the factors influencing PrEP-prescribing behaviors among PCPs in the Southern US. Here, the key results are interpreted, and their implications for improving the PrEP uptake in this high-risk region are discussed.

The study revealed that race and practice setting are significant predictors of PrEP-prescribing behaviors. Non-White PCPs and those practicing in urban and suburban settings were more likely to be in advanced stages of change and to prescribe PrEP. This suggests that PCPs from diverse racial backgrounds and those in urbanized areas may have better access to resources and training related to PrEP or may be more attuned to the needs of high-risk populations. Tailored educational and support programs targeting PCPs in rural areas and those from less diverse backgrounds are crucial. Such initiatives could help bridge the gap in PrEP-prescribing practices and ensure more uniform access to PrEP across different regions.

Receiving specific training on PrEP significantly predicted higher stages of change and increased likelihood of PrEP prescription. This underscores the importance of education in equipping PCPs with the knowledge and confidence to prescribe PrEP. Integrating PrEP training into medical school curricula and continuing medical education programs is essential. Training programs should focus on practical aspects of PrEP prescription, including patient counseling, risk assessment, and adherence strategies.

The study found that perceived barriers such as lack of provider training, low adherence likelihood, and potential HIV resistance negatively impacted PrEP prescribing. Conversely, facilitators such as access to resources, streamlined insurance procedures, and patient motivation positively influenced prescribing behaviors. Addressing provider concerns through evidence-based information and practical solutions is key. For instance, providing data on PrEP adherence rates and addressing misconceptions about HIV resistance can alleviate provider hesitations. Ensuring easy access to prescribing resources and simplifying insurance processes can further support PCPs in prescribing PrEP.

PCPs who reported seeing a higher proportion of African American patients were more likely to be in advanced stages of PrEP prescribing and to have prescribed PrEP. This aligns with the higher HIV burden among African American communities in the Southern US and indicates a responsive adaptation by PCPs to the needs of their patient populations. Public health initiatives should focus on areas with high African American populations to enhance PrEP awareness and access. Collaborative efforts between community organizations and healthcare providers can facilitate targeted outreach and support.

The study highlighted systemic barriers such as lack of insurance coverage and financial constraints as significant impediments to PrEP uptake. These barriers were rated highly by PCPs, indicating their critical role in limiting access to PrEP. Policy changes that expand insurance coverage for PrEP and reduce out-of-pocket costs are vital. Advocacy efforts should focus on removing financial barriers and ensuring that PrEP is accessible to all individuals at risk of HIV, regardless of their socioeconomic status.

Understanding the landscape: barriers and facilitators to PrEP uptake in the Southern United States

The Southern US presents a unique and challenging landscape for HIV prevention, particularly through the utilization of PrEP. Despite the high efficacy of PrEP in reducing HIV transmission, its uptake remains critically low in this region, especially among African American communities who are disproportionately affected by HIV. This study sheds light on several factors that contribute to the low uptake of PrEP and offers insights into potential interventions to address these barriers.

PCPs play a pivotal role in the dissemination and prescription of PrEP. However, many PCPs in the Southern US are not adequately informed or trained about PrEP, its benefits, and its prescribing guidelines. This lack of knowledge creates a significant barrier to the PrEP uptake. Studies have shown that providers who are unaware of PrEP or its efficacy are less likely to discuss it with patients or prescribe it, thereby limiting access to this vital preventive measure.

The stigma related to HIV and PrEP is a pervasive issue that affects both patients and healthcare providers. PCPs may hold stigmatizing views towards individuals at high risk of HIV, such as African American MSM and transgender individuals, which can hinder their willingness to prescribe PrEP. This stigma not only impacts the provider's behavior but also discourages patients from seeking PrEP due to fear of judgment and discrimination.

The demanding nature of primary care practice, characterized by high patient volumes and limited consultation times, often results in preventive health discussions, including PrEP, being deprioritized. Discussing PrEP requires a detailed conversation about HIV risk behaviors, medication adherence, and potential side effects, and many PCPs feel that they do not have adequate time to cover it.

Systemic barriers such as lack of health insurance coverage, high out-of-pocket costs for PrEP, and cumbersome insurance prior authorization procedures further impede the uptake of PrEP. Patients who cannot afford PrEP or navigate the complexities of insurance may be unable to access this preventive measure, despite its potential to significantly reduce HIV transmission.

Enhancing the knowledge and skills of PCPs through targeted training programs can significantly improve PrEP-prescribing practices. Education that covers PrEP efficacy, prescribing guidelines, and patient counseling can empower PCPs to incorporate PrEP discussions into their routine practice. Integrating PrEP services into routine primary care can normalize its use and reduce stigma. This can be achieved by incorporating PrEP discussions into standard HIV-screening protocols and utilizing electronic health record prompts to remind providers to discuss PrEP with eligible patients.

Community-based interventions that involve partnerships between public health departments, federally qualified health centers, and community organizations can enhance access to PrEP. These collaborations can facilitate outreach and education efforts, particularly in underserved and high-risk communities. Advocating for policy changes that reduce financial barriers to PrEP, such as expanded insurance coverage and streamlined prior authorization processes, is crucial. Additionally, ensuring that PrEP is included in medical school curricula and continuing medical education programs can address the knowledge gap among healthcare providers.

Advancing health equity through targeted PrEP-prescribing strategies

The findings from this study provide valuable insights into how addressing the identified barriers and facilitators of PrEP-prescribing behaviors among PCPs can promote health equity. By highlighting the role of race and practice settings as significant predictors of PrEP prescribing, the study underscores the necessity of tailoring educational and support programs to enhance PrEP access in rural areas and among less diverse PCPs. These targeted interventions can help bridge the gap in PrEP-prescribing practices, ensuring more uniform access across different regions and demographics. Furthermore, by focusing on systemic barriers such as the lack of health insurance coverage and financial constraints, the study emphasizes the need for policy changes to expand access to PrEP. These changes are crucial for ensuring that all individuals at risk of HIV, regardless of socioeconomic status, have access to PrEP. Through these efforts, public health initiatives can work toward reducing disparities in HIV prevention and enhancing health equity in the Southern US, particularly among high-risk African American communities. Collaborative efforts involving community organizations and PCPs can further facilitate targeted outreach and support, ultimately contributing to a more equitable healthcare landscape.

Applying the TTM to PrEP prescribing

The TTM offers a robust framework for understanding and predicting PrEP-prescribing behaviors among PCPs. By identifying where PCPs are in their readiness to prescribe PrEP, targeted interventions can be designed to move them through the stages of change towards action and maintenance. For instance, PCPs in the precontemplation stage may benefit from educational campaigns that raise awareness about the high HIV burden in the Southern US and the effectiveness of PrEP. Those in the contemplation stage might require detailed information on how to discuss PrEP with patients and address common concerns. PCPs in the preparation stage could benefit from practical training sessions that build their confidence and competence in prescribing PrEP.

Limitations

While this study provides valuable insights into the factors influencing PrEP-prescribing behaviors among primary care providers in the Southern United States, several limitations must be considered when interpreting the findings. These limitations pertain to the sample size, recruitment methods, study design, potential biases, and contextual factors. Understanding these limitations is crucial for accurately assessing the study's contributions and for guiding future research in this area.

Sample Size

The sample size for this study was determined to be 219 participants based on a power analysis to achieve a medium effect size with an alpha of .05 and a power of .80. However, the final dataset included 223 participants after removing incomplete responses. While this sample size was adequate for the statistical analyses performed, it may not fully capture the diversity and variability of primary care providers' experiences and perspectives across the entire Southern US region. The relatively small sample size limits the generalizability of the findings and may overlook nuanced differences within subgroups of the population.

Limitations of Internet Recruitment

Participants were recruited using social media and email due to COVID-19 lockdowns, which may have introduced selection bias. Internet recruitment often leads to a sample that is more technologically savvy and possibly more informed about current health trends and practices compared to the general population of PCPs. This method might also exclude those without regular internet access or those who are less inclined to engage with online platforms, potentially skewing the results towards more proactive and engaged practitioners.

Cross-Sectional Study Design

The cross-sectional design of this study captures a snapshot of primary care providers' PrEP-prescribing behaviors and attitudes at a single point in time. While this provides valuable insights, it does not allow for the assessment of changes over time or the establishment of causal relationships. Longitudinal studies would be more appropriate to observe trends, changes in behavior, and the impact of interventions over time. Additionally, qualitative and mixed methods studies could be utilized to explore the underlying reasons for providers' behaviors and attitudes, providing a deeper understanding of the contextual factors influencing PrEP-prescribing practices.

Reporting Bias

Self-reported data are inherently susceptible to reporting bias. Participants might have provided socially desirable responses, especially on sensitive topics like stigma and perceived barriers to PrEP prescribing. The anonymity of the survey was intended to mitigate this, but it cannot completely eliminate the possibility of biased reporting. Additionally, recall bias may affect the accuracy of responses related to past behaviors and experiences.

Other limitations

Generalizability

The study focused on PCPs in 10 Southern states, which limits the generalizability of the findings to other regions. The specific cultural, socioeconomic, and healthcare landscape of the Southern US may not be applicable to other areas, and thus the results should be interpreted with caution when considering broader applications.

Measurement Limitations

The survey instrument was adapted from the HIV PrEP survey used by Terndrup et al. (2019) and included the transtheoretical model staging algorithm. Although permission was obtained to use the survey, the validity and reliability of the instrument were not established for this specific study. Therefore, the measurements may not accurately reflect the constructs they were intended to assess.

Impact of COVID-19

The timing of the study during the COVID-19 pandemic may have influenced the responses. The pandemic significantly altered healthcare practices, priorities, and resource allocation, which could have affected PCPs' attitudes and behaviors towards PrEP prescribing. The long-term effects of these changes remain to be seen and should be considered in future research.

These limitations highlight the need for further research with larger, more diverse samples, longitudinal designs, and mixed methods approaches to fully understand and address the barriers and facilitators to PrEP prescribing among primary care providers in the Southern United States.

## Conclusions

In conclusion, addressing the barriers to PrEP uptake in the Southern US necessitates a comprehensive and multifaceted strategy that encompasses enhancing PCPs' knowledge and skills, reducing stigma, improving systemic support, and fostering community-based interventions. To achieve this, it is imperative to develop comprehensive training programs that focus on the latest PrEP guidelines, risk assessment, and effective communication strategies. These programs should be accessible through various platforms, including workshops, online modules, and CME courses, facilitated by experts in HIV prevention and sexual health. Ensuring these training opportunities are provided by reputable organizations in settings such as healthcare conferences, clinics, and medical schools will ensure their accessibility for both current and future healthcare professionals. Community-based interventions will play a crucial role in leveraging local resources and cultural contexts to increase PrEP uptake. By partnering with local community centers, faith-based organizations, and advocacy groups, educational outreach and stigma-reduction campaigns can be effectively implemented. Initiatives such as peer-led support groups, PrEP navigation services, and mobile health clinics are instrumental in increasing awareness about and access to PrEP. Tailoring these interventions to the specific needs of the community and incorporating input from local stakeholders are essential to ensure their relevance and effectiveness. Finally, employing frameworks like the TTM allows for the development of targeted strategies that effectively increase the PrEP uptake and reduce HIV transmission in this high-risk region. The findings from this study highlight the critical need for tailored interventions that address the unique challenges faced by PCPs and patients in the Southern US. Ultimately, these efforts will contribute to better health outcomes and greater health equity, underscoring the importance of a coordinated approach in tackling the barriers to PrEP uptake in this region.

## Appendices

Instrument

Informed Consent

University of Missouri Sinclair School of Nursing

Title of study: Primary Care Provider PrEP Prescribing Practices: Southern United States

Investigators(s): Daryl Traylor, PhD (c). For questions or concerns about the study, you may contact Daryl Traylor at 480-482-0740 or via email dotcf2@mail.missouri.edu.

Purpose of the study

You are invited to participate in this research study. The purpose of this research study is to learn about primary care provider (PCP) training experiences and knowledge, attitudes, and behaviors regarding prescribing HIV pre-exposure prophylaxis (“PrEP”) for African Americans residing in the Southern United States. This anonymous survey should take approximately 10 to 15 minutes to complete.

Participants

You are being asked to participate in this study because you fit these criteria: (1) You are a licensed and practicing physician who has completed residency training, a nurse practitioner, or a physician assistant. (2) You practice primary care medicine. (3) Your medical practice is located in one or more of the following states: Alabama, Arkansas, Georgia, Florida, Louisiana, Mississippi, North Carolina, South Carolina, Tennessee, or Texas.

Procedures

If you volunteer to participate in this study, you will be asked to do the following: Complete an online survey using the Qualtrics platform.

Benefits of participation

There will be no direct benefits to you as a participant in this study. However, I hope to learn more about the HIV PrEP prescribing practices of primary care as they pertain to African Americans in the Southern United States. I hope that the results will then be used to improve primary care provider HIV PrEP prescribing practices for African Americans residing in Southern United States.

Risks of participation

There are minimal anticipated risks in this study. Personal questions are asked, which may make you feel uncomfortable. However, the survey does not ask you to provide identifying information such as name, address, email address, and phone number. Your answers will not be readily linked to you. In addition, all results will be reported as a group.

Cost/compensation

There will be no financial cost to you to participate in this study. The study will take about 10-15 minutes of your time.

Confidentiality

All information gathered in this study will be kept anonymous. As stated earlier, no reference will be made in written or oral materials that could link you to this study. All records will be stored on a password protected, encrypted hard drive for 3 years after completion of the study. After the storage time, the information gathered will be destroyed as appropriate by me.

Voluntary participation

Your participation in this study is voluntary. You may refuse to participate in this study or in any part of this study. You may withdraw at any time. You are encouraged to ask questions about this study at the beginning or any time during the research study.

You may contact the University of Missouri Institutional Review Board (IRB) if you:

Have any questions about your rights as a study participant;

Want to report any problems or complaints; or

Feel under any pressure to take part or stay in this study.

The IRB is a group of people who review research studies to make sure the rights of participants are protected. Their phone number is (573) 882-3181.

Participant Consent

I have read the above information and agree to participate in this study. I have been able to ask questions about the research study. I am at least 18 years of age.

Do you consent to participate in this study?

Yes

No

The next set of questions will ask you about your eligibility for this study. Thank you for your responses.

Are you 18 years of age or older?

Yes

No

Are you a licensed physician, Advanced Practice Registered Nurse, or Physician Assistant?

Yes

No

Do you currently practice primary care medicine?

Yes

No

In which of the following states do you practice primary care medicine in? Check all that apply:

Alabama

Arkansas

Georgia

Florida

Louisiana

Mississippi

North Carolina

South Carolina

Tennessee

Texas

None of the above

Block 1

PrEP stands for “Pre-Exposure Prophylaxis”, the use of any medicine to prevent a disease before exposure to that disease. For the purposes of this survey, the term refers to the use of an oral antiretroviral medication taken on a daily basis by people at high risk of exposure to HIV to prevent HIV infection. Truvada, a combination of two antiretroviral medications tenofovir disoproxil fumarate and emtricitabine, was approved for PrEP in 2012 by the FDA and recommended in 2014 by the CDC. Descovy, a combination of emtricitabine and tenofovir alafenamide, was approved by the FDA in 2019. Any healthcare provider who is authorized to write prescriptions can write a prescription for PrEP. You do not have to be an HIV specialist or infectious disease specialist to write a prescription for PrEP.

1.     Do you regularly write prescriptions for PrEP for your African American patients who have PrEP indications?

Yes, I have been for less than 6 months.

No, but I intend to in the next 30 days.

No, but I intend to in the next 6 months.

No, and I do not intent to in the next 6 months.

Block 2

Listed below are several possible facilitators of prescribing PrEP. How important is each of these facilitators to you in deciding whether or not to prescribe PrEP for your African American patients who have PrEP indications?

1.     How important are the following in your decision to prescribe PrEP?

**Table 10:** Facilitators of PrEP prescribing PrRP, pre-exposure prophylaxis

	1. Not important	2. Slightly important	3. Moderately important	4. Very important	5. Extremely important
PrEP training during residency	○	○	○	○	○
Staff or providers in your clinic who are knowledgeable about PrEP provision	○	○	○	○	○
Access to resources such as PrEP prescription guidelines and protocols	○	○	○	○	○
On-site support (i.e., risk reduction or adherence counselors, social workers)	○	○	○	○	○
Clinic in-service PrEP training	○	○	○	○	○
Knowledge of PrEP's efficacy	○	○	○	○	○
Patient motivation or "buy in" to consistently and properly use PrEP as prescribed	○	○	○	○	○
Peers who prescribe PrEP	○	○	○	○	○
Patient access to financial incentives that would lower the cost of PrEP	○	○	○	○	○
Streamlined insurance prior authorization procedures	○	○	○	○	○

Listed below are several possible barriers of prescribing PrEP. How important is each of these barriers to you in deciding whether or not to prescribe PrEP for your African American patients who have PrEP indications?

**Table 11:** Barriers to PrEP prescribing PrRP, pre-exposure prophylaxis

	1. Not important	2. Slightly important	3. Moderately important	4. Very important	5. Extremely important
Lack of provider training/education regarding PrEP	○	○	○	○	○
Lack of clinic leadership support regarding PrEP	○	○	○	○	○
Lack of clinical guidelines/protocols for prescribing/monitoring PrEP	○	○	○	○	○
Clinic and lab monitoring requirements (e.g., seeing patient and obtaining HIV tests and STI screening every 3 months; checking renal function every 6 months)	○	○	○	○	○
Staffing time constraints related to risk reduction and PrEP adherence counseling	○	○	○	○	○
Lack of insurance coverage and out-of-pocket patient costs for PrEP and related care (e.g., lab work)	○	○	○	○	○
Likelihood of low adherence to PrEP	○	○	○	○	○
Likelihood of HIV resistance developing	○	○	○	○	○
Patients may engage in riskier behavior while on PrEP	○	○	○	○	○
Insufficient evidence of PrEP's effectiveness	○	○	○	○	○

Block 3

The following questions are about your current personal and practice demographics.

1.     What is your age?

2.     What is your gender?

Male

Female

Gender queer/Non-conforming

Transgender

Prefer not to answer

3.     With respect to sexual orientation, how do you self-identify?

Heterosexual

Gay

Lesbian

Bisexual

Other

Prefer not to answer

4.     With respect to race, how do you self-identify (select all that apply)?

Asian or Asian American

Black or African American

Native Hawaiian and Pacific Islander

White

Multiracial

Other

Prefer not to answer

5.     Do you identify as being Hispanic or Latino?

Yes

No

6.     I am a...

Physician

Advanced Practice Registered Nurse

Physician Assistant

7.     What is your highest degree completed?

Doctor of Osteopathic Medicine (D.O.)

Medical Doctor (M.D.)

Master of Science - Nursing (MSN) or equivalent

Doctor of Nursing Practice (DNP)

Master of Science - Physician Assistant (MSPA) or equivalent

Doctor of Medical Science - Physician Assistant (DMSPA) or equivalent

8.     How would you classify the geographic area you practice in?

Urban - The U.S. Census Bureau defines an urban area as an area with 50,000 or more people.

Suburban - The U.S. Census Bureau defines suburban areas as those areas that lie on the fringes of urban areas and are in easy commuting distance of urban areas.

Rural - The U.S. Census Bureau defines a rural area as any area outside of urban and suburban areas with a population of 0 to 49,999 people.

9.     What is the zip code of your primary care practice?

10.     How many years have you practiced primary care healthcare?

11.     For each racial/ethnic category, please mark the percentage of each group that makes up your clinic patient population. Please give your best estimate.

**Table 12:** Clinic patient population

	African American	White	American Indian/Alaska Native	Latinx	Asian	Native Hawaiian or Pacific Islander
0%	○	○	○	○	○	○
1%-25%	○	○	○	○	○	○
26%-50%	○	○	○	○	○	○
51%-75%	○	○	○	○	○	○
76%-100%	○	○	○	○	○	○

12.     Do you routinely screen your African American patients for HIV?

Yes

No

13.     Have you ever written a prescription for PrEP for an African American patient?

Yes

No

14.     Do you routinely write PrEP prescriptions for your African American patients?

Yes

No

15.     Have you ever referred an African American patient for a PrEP prescription (e.g., to a PrEP provider or Infectious Disease/HIV clinic)? 

Yes

No

16.     Do you routinely refer African American patients for PrEP prescriptions (e.g., to a PrEP provider or Infectious Disease/HIV clinic)?

Yes

No

17.     Before this survey, had you ever heard of PrEP?

Yes

No

18.     Have you received specific training on PrEP?

Yes

No

Thank you for completing this survey!
